# New Insights into the Bacterial Targets of Antimicrobial Blue Light

**DOI:** 10.1128/spectrum.02833-22

**Published:** 2023-02-21

**Authors:** Carolina dos Anjos, Leon G. Leanse, Martha S. Ribeiro, Fábio P. Sellera, Milena Dropa, Victor E. Arana-Chavez, Nilton Lincopan, Maurício S. Baptista, Fabio C. Pogliani, Tianhong Dai, Caetano P. Sabino

**Affiliations:** a Wellman Center for Photomedicine, Massachusetts General Hospital, Harvard Medical School, Boston, Massachusetts, USA; b Department of Internal Medicine, School of Veterinary Medicine and Animal Science, University of São Paulo, São Paulo, Brazil; c University of Gibraltar, Europa Point Campus, Gibraltar; d Center for Lasers and Applications, Nuclear and Energy Research Institute (IPEN-CNEN), São Paulo, Brazil; e School of Veterinary Medicine, Metropolitan University of Santos, Santos, Brazil; f MicroRes Laboratory, School of Public Health, University of São Paulo, São Paulo, Brazil; g Department of Biomaterials and Oral Biology, University of São Paulo, São Paulo, Brazil; h Department of Clinical and Toxicological Analysis, School of Pharmaceutical Sciences, University of São Paulo, São Paulo, Brazil; i Department of Microbiology, Institute for Biomedical Sciences, University of São Paulo, São Paulo, Brazil; j Department of Biochemistry, Institute of Chemistry, University of São Paulo, São Paulo, Brazil; k Biolambda, Scientific and Commercial Ltd., São Paulo, Brazil; Emory University School of Medicine

**Keywords:** endogenous chromophores, lipid peroxidation, membrane permeabilization, protein carbonylation, reactive oxygen species

## Abstract

Antimicrobial blue light (aBL) offers efficacy and safety in treating infections. However, the bacterial targets for aBL are still poorly understood and may be dependent on bacterial species. Here, we investigated the biological targets of bacterial killing by aBL (λ = 410 nm) on three pathogens: Staphylococcus aureus, Escherichia coli, and Pseudomonas aeruginosa. Initially, we evaluated the killing kinetics of bacteria exposed to aBL and used this information to calculate the lethal doses (LD) responsible for killing 90 and 99.9% of bacteria. We also quantified endogenous porphyrins and assessed their spatial distribution. We then quantified and suppressed reactive oxygen species (ROS) production in bacteria to investigate their role in bacterial killing by aBL. We also assessed aBL-induced DNA damage, protein carbonylation, lipid peroxidation, and membrane permeability in bacteria. Our data showed that P. aeruginosa was more susceptible to aBL (LD_99.9_ = 54.7 J/cm^2^) relative to S. aureus (LD_99.9_ = 158.9 J/cm^2^) and E. coli (LD_99.9_ = 195 J/cm^2^). P. aeruginosa exhibited the highest concentration of endogenous porphyrins and level of ROS production relative to the other species. However, unlike other species, DNA degradation was not observed in P. aeruginosa. Sublethal doses of blue light (<LD_90_) could damage the cell membrane in Gram-negative species but not in S. aureus. In all bacteria, oxidative damage to bacterial DNA (except P. aeruginosa), proteins, and lipids occurred after high aBL exposures (>LD_99.9_). We conclude that the primary targets of aBL depend on the species, which are probably driven by variable antioxidant and DNA-repair mechanisms.

**IMPORTANCE** Antimicrobial-drug development is facing increased scrutiny following the worldwide antibiotic crisis. Scientists across the world have recognized the urgent need for new antimicrobial therapies. In this sense, antimicrobial blue light (aBL) is a promising option due to its antimicrobial properties. Although aBL can damage different cell structures, the targets responsible for bacterial inactivation have still not been completely established and require further exploration. In our study, we conducted a thorough investigation to identify the possible aBL targets and gain insights into the bactericidal effects of aBL on three relevant pathogens: Staphylococcus aureus, Escherichia coli, and Pseudomonas aeruginosa. This research not only adds new content to blue light studies but opens new perspectives to antimicrobial applications.

## INTRODUCTION

The uncontrolled spread of antimicrobial-resistant pathogens, along with delays in the discovery of novel antibiotics, has been responsible for a global public health crisis (1–3). It is estimated that antibiotic-resistant bacterial infections have been responsible for more than 1.27 million deaths worldwide in 2019 (2). As a matter of urgency, the World Health Organization (WHO) has encouraged studies focused on the discovery, research, and development of new approaches to face this unbridled threat (3).

Light-based therapies are considered promising approaches for the treatment of a myriad of diseases, including those caused by infectious agents. Although the use of light to fight infections seems to be a futuristic advance, Niels Ryberg Finsen was awarded the Nobel Prize for his contribution in using light for the treatment of infectious diseases in 1903 (4). Several recent studies have demonstrated the effectiveness of antimicrobial blue light (aBL) against a vast array of pathogens, including bacteria, algae, yeasts, and molds (5, 6). In addition, aBL has been effective against multidrug-resistant pathogens, and has also shown to be highly compatible and synergistic when applied concurrently with traditional and nontraditional antimicrobial agents (7–10). Importantly, aBL is highly selective in killing microbial pathogens over human cells, does not promote adverse effects, and the selection of resistant strains is unlikely. This makes aBL an attractive approach for clinical use (5, 6).

To date, the most accepted hypothesis is that aBL elicits its antimicrobial effects as a result of photoexcitation of endogenous chromophores (e.g., porphyrins and/or flavins) to produce cytotoxic reactive oxygen species (ROS), which trigger cellular damage to lipids and proteins (11–13). It was also suggested that a flavin photoproduct, lumichrome, may play a role as an endogenous photosensitizer that is excitable by shorter aBL wavelength (14).

Indeed, although the bacterial targets of aBL have not been fully elucidated, studies have suggested an increase in membrane permeability due to lipid peroxidation with a consequent decrease in unsaturated fatty acids (11–13). More recently, Walker and collaborators reported a two-phase protein damage/oxidative stress response to aBL in Campylobacter jejuni, a foodborne zoonotic pathogen (12). The authors showed that there is a specific regulation of genes that encode proteins involved in the cellular response to global protein damage, which depends on the light dose, i.e., whether it is bacteriostatic or bactericidal.

More than a century has gone by since Finsen's discovery, but the bacterial targets of aBL have yet to be fully elucidated. Therefore, in this study, we performed a robust investigation to determine the effects of aBL against bacteria of clinical interest, such as Escherichia coli (Gram-negative), Pseudomonas aeruginosa (Gram-negative), and Staphylococcus aureus (Gram-positive). First, we obtained the killing kinetics for bacteria exposed to aBL. We measured porphyrin content and imaged bacterial cells by fluorescence lifetime imaging microscopy (FLIM) to evaluate the spatial distribution of porphyrin content in the absence of aBL. Next, we quantified ROS formation under aBL exposure and used ROS scavengers to examine the role of singlet oxygen and hydroxyl radicals in aBL bacterial killing. To observe potential bacterial targets of aBL, we performed transmission electron microscopy (TEM). To confirm bacterial targets, we assessed DNA damage, protein carbonylation, lipid peroxidation, and membrane permeability, by comparing the three bacterial species following aBL exposure.

## RESULTS

### aBL effectively killed bacteria, albeit with variable efficacies.

**FIG 1:**
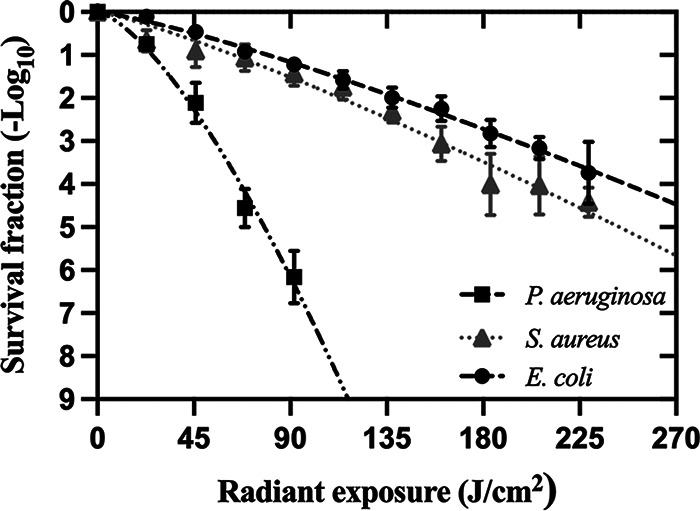
Dose-response curves for E. coli, P. aeruginosa and S. aureus exposed to aBL (λ = 410 ± 10 nm). Values are presented as means ± SEM.

The killing curve of aBL against P. aeruginosa, S. aureus, *and* E. coli is exhibited in Fig. 1. For LD_90_, we observed that P. aeruginosa is significantly more susceptible than S. aureus (*P* = 0.001) and E. coli (*P* < 0.0001) to aBL. Indeed, the LD_90_ and LD_99.9_ for P. aeruginosa were 25.5 and 54.7 J/cm^2^, respectively. In contrast, S. aureus and E. coli were equally tolerant to aBL exposure (*P* = 0.15). Radiant exposures of 63.5 and 158.9 J/cm^2^ were required to achieve the LD_90_ and LD_99.9_ for S. aureus, respectively. For E. coli, LD_90_ and LD_99.9_ were 79.6 and 195 J/cm^2^, respectively.

### TEM revealed significant aBL-induced ultrastructural changes in all bacteria.

**FIG 2:**
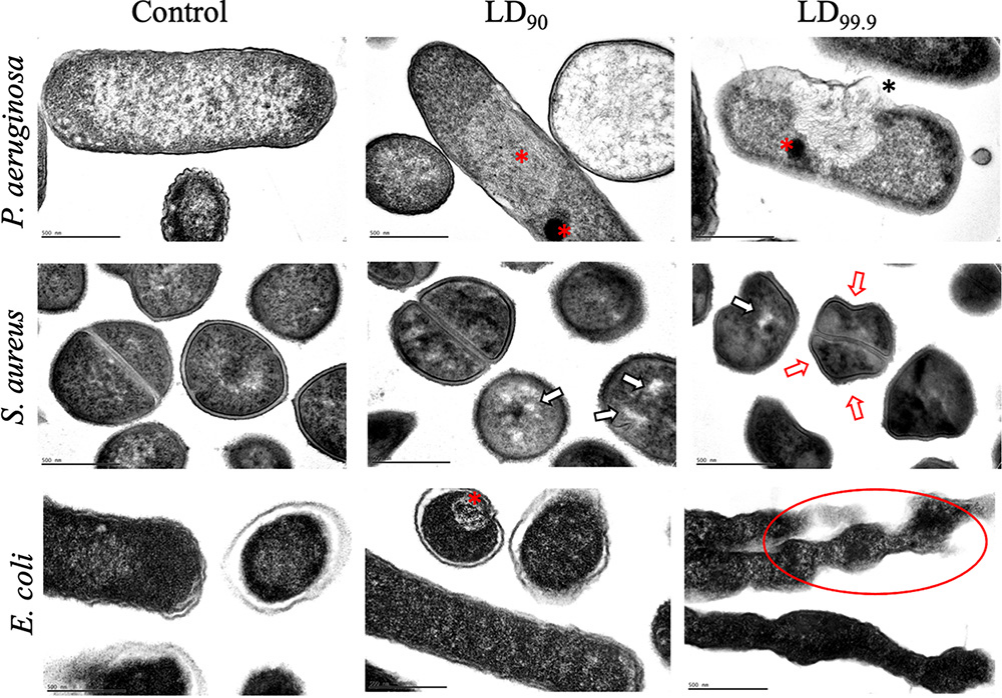
Representative transmission electron micrographs illustrating aBL-induced ultrastructural damages in P. aeruginosa, S. aureus, and E. coli for LD_90_ and LD_99.9_. Red asterisk, agglutination of intracellular contents; black asterisk, cell wall/membrane damage; white arrow, leakage of intracellular contents; red arrow, membrane destabilization; red circle, elongation and morphological irregularities. Bars: 500 nm.

Ultrastructural changes in P. aeruginosa, S. aureus, *and* E. coli exposed to LD_90_ and LD_99.9_ of aBL are shown in Fig. 2 and Fig. S1 to 3. For P. aeruginosa exposed to LD_90_, we observed a dense formation in the cytoplasm that suggests an agglutination of intracellular contents. When exposed to LD_99.9_, we observed pronounced damages in the cell wall/membrane as well as cytosol leakage. Regarding S. aureus, there was vacuole formation following LD_90_, indicating cytoplasmic damage. Increasing the aBL exposure to achieve the LD_99.9_, conformational changes were perceived in the cell wall/membrane. For E. coli, evident aggregation of cellular content and detached membrane structures were observed. Besides that, the LD_99.9_ promoted clear morphological changes, such as elongation and irregularities, suggesting damage to the cell wall/membrane.

### Porphyrins were present in all bacteria tested.

**FIG 3:**
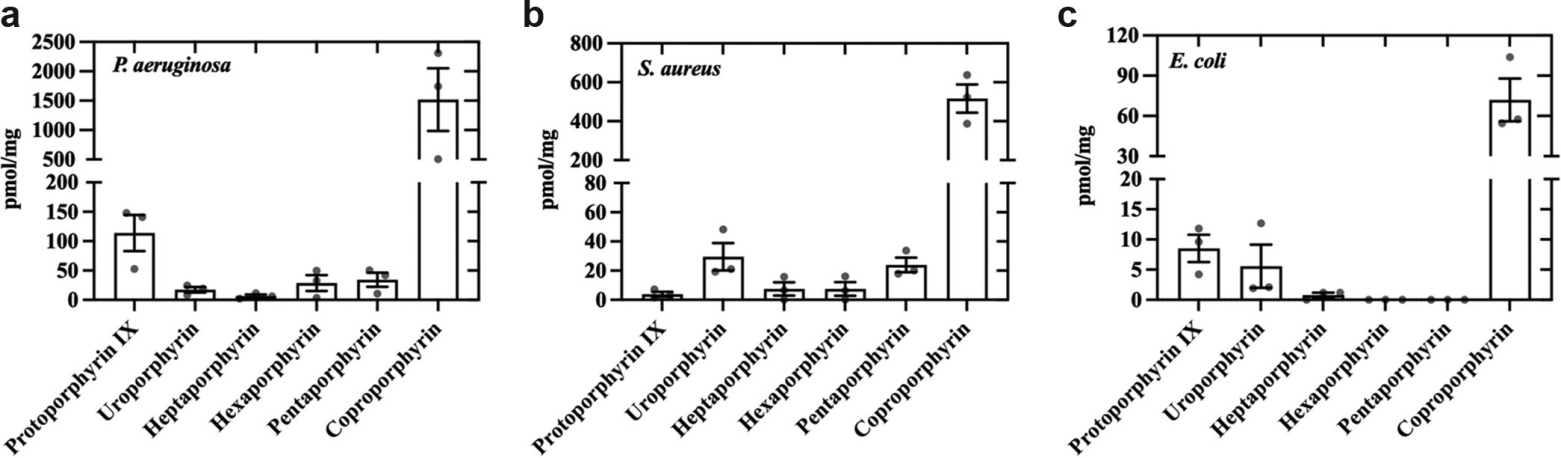
Porphyrin concentrations present within (a) P. aeruginosa, (b) S. aureus, and (c) E. coli. Symbols (•) represent the mean of three technical replicates. Data are presented as scatterplots with bar ± SEM.

We observed that all species presented significant concentrations of porphyrins (Fig. 3), with coproporphyrin being the most abundant intracellular porphyrin regardless of the species. P. aeruginosa showed the highest concentrations (1,606.5 ± 564.06 pmol/mg), followed by S. aureus (516.0 ± 72.4 pmol/mg) and E. coli (71.9 ± 15.9 pmol/mg). P. aeruginosa also exhibited a moderate amount of protoporphyrin IX (113.8 ± 30.68 pmol/mg) while uroporphyrin was the second most abundant in S. aureus (29.5 ± 9.38 pmol/mg). Indeed, the porphyrin content of E. coli was much lower than that observed for P. aeruginosa and S. aureus. It is important to note that for this assay, all bacterial species were in the stationary phase of growth, because growth conditions likely affect bacteria physiology and porphyrin content may be condition-dependent.

**FIG 4:**
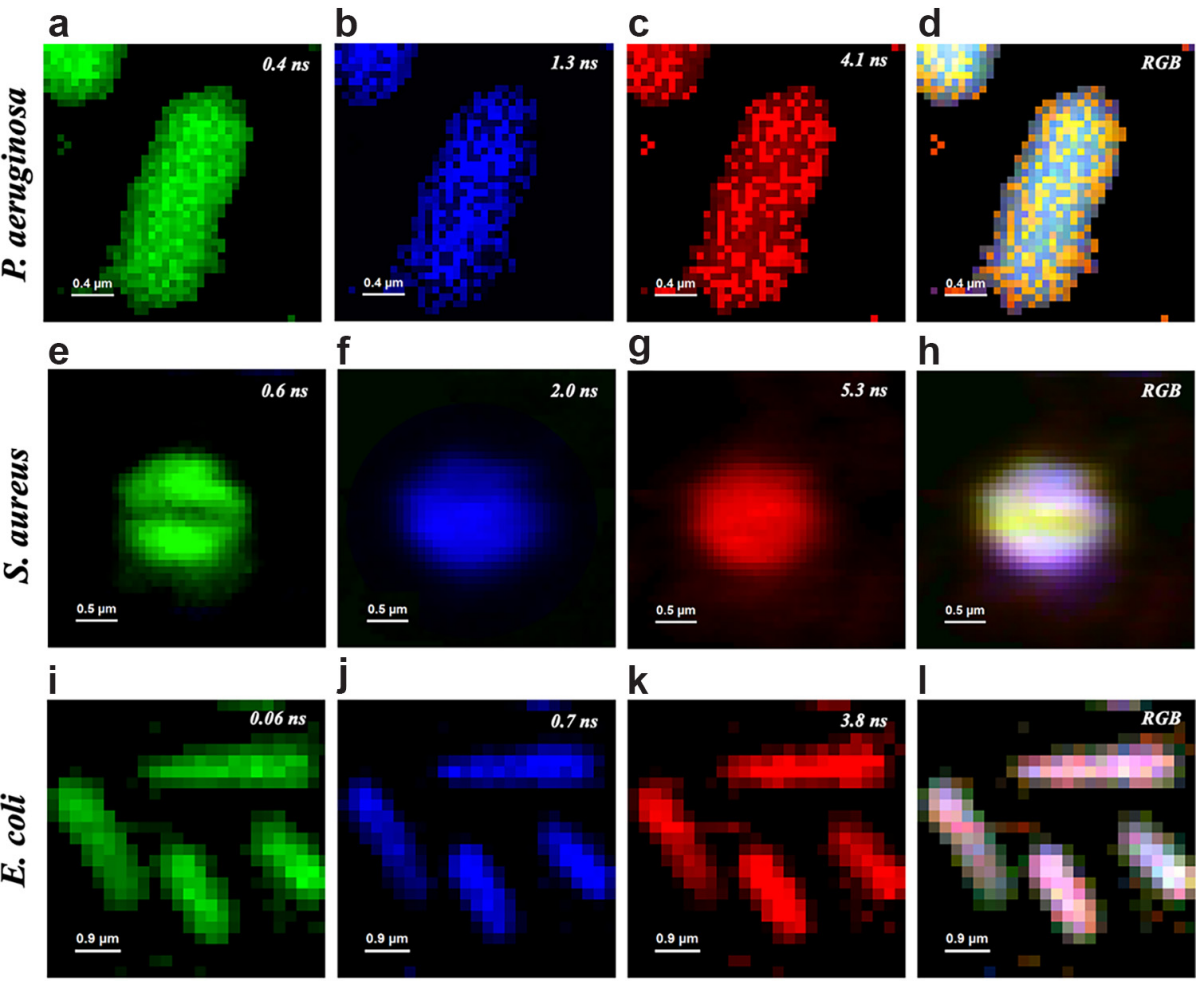
FLIM images of endogenous fluorophores for P. aeruginosa (a to d), S. aureus (e to h), and E. coli (i to l). Different colors represent different fluorescence lifetimes for each bacterial species (green < blue < red). Homogeneous distribution of fluorophores can be observed for each lifetime decay. Shorter lifetimes are observed for E. coli. No fluorophores were detected in the septum of S. aureus for the shortest lifetime (e). Merged fluorescence signal (RGB) in each bacterial species (d, h, l) is also depicted.

The spatial distribution profile of intrinsic fluorophores in S. aureus, E. coli, and P. aeruginosa evaluated by FLIM are displayed in Fig. 4. FLIM is a unique technique that allows mapping the spatial localization of endogenous fluorophores and their interaction with biomolecules due to lifetime decay. We observed fluorophores homogeneously distributed throughout the cell due to the lack of compartmentalization of bacteria. Different fluorescence lifetimes for each bacterial species, represented by different colors, indicate either the presence of more than one fluorophore or different timespan interactions of fluorophores with intracellular components. Noteworthily, shorter fluorescence lifetimes were detected for E. coli regardless of the fluorophore or its interaction inside the cell. Besides, the septum dividing S. aureus cells appears free of fluorophores or interaction of short lifetime decay (0.6 ns) while others were detected in longer lifetimes.

### aBL resulted in significant ROS production in all bacteria.

**FIG 5:**
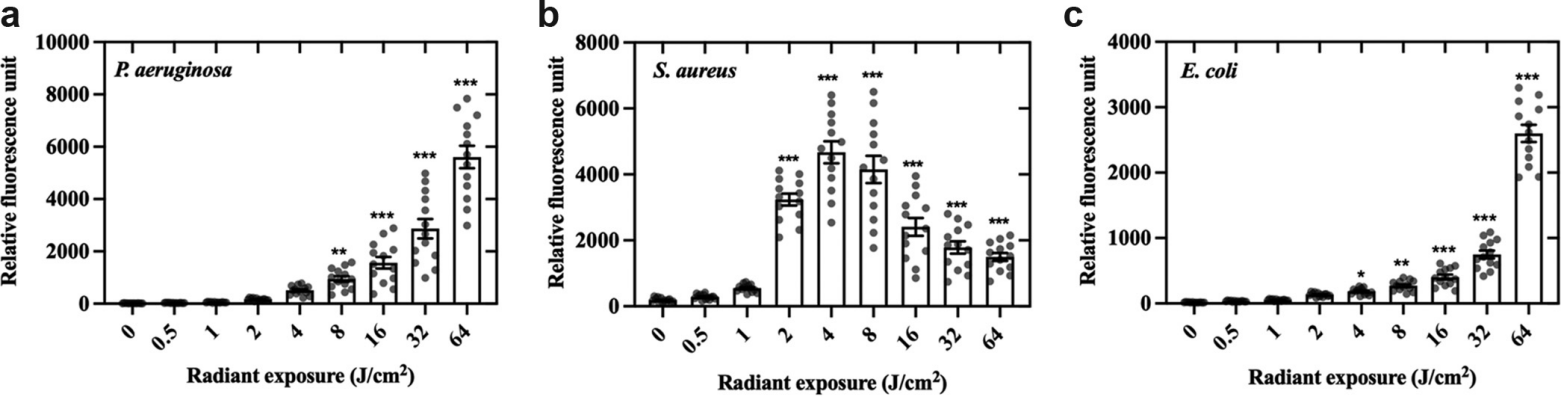
Relative fluorescence units proportional to intracellular ROS production in P. aeruginosa (a), S. aureus (b), and E. coli (c) after aBL exposure. Symbols (•) represent the average of cumulative measurements over 1 h. Data are presented as scatterplots with bar ± SEM. *, *P* < 0.05; **, *P* < 0.01; ***, *P* < 0.001 compared to unirradiated control.

We quantified ROS production as a consequence of different aBL exposures for P. aeruginosa, S. aureus, and E. coli by the relative fluorescence units (RFU) of oxidized DCFH-DA, which is proportional to ROS production (Fig. 5). Noteworthily, we were unable to measure ROS for P. aeruginosa in the absence of aBL, even though this species synthesizes pyocyanin, a redox-active compound that could react with DCFH-DA (15). Besides, P. aeruginosa and E. coli showed exponential growth of RFU dependent on the increase of the aBL radiant exposure. Indeed, the quantity of ROS was approximately 2-fold higher for P. aeruginosa than E. coli following 64 J/cm^2^ (5,606 ± 308 × 2,617 ± 128, respectively). Curiously, the behavior is completely different for S. aureus. In this case, ROS production reaches a peak at 4 J/cm^2^ (4,670 ± 486) and gradually decreases until 64 J/cm^2^ (1498 ± 80).

### Hydroxyl radicals and singlet oxygen played a role in aBL effect.

**FIG 6:**
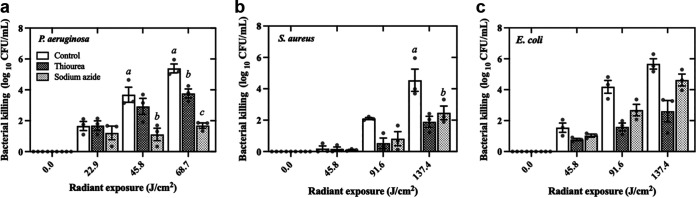
Effects of ROS scavengers on aBL killing of bacteria. Sodium azide (10 mM) and thiourea (150 μM) were used as scavengers of singlet oxygen and hydroxyl radicals, respectively. (a) P. aeruginosa, (b) S. aureus, and (c) E. coli. Symbols (•) represent the mean of three technical replicates. Data are presented as scatterplots with bar ± SEM. Different letters indicate significant differences between treatments (*P* < 0.05).

Azide (N_3_-) is an efficient physical quencher of ^1^O_2_, which is used as a reference compound to measure the oxidation kinetics of singlet oxygen (16). On the other hand, thiourea is an effective scavenger of OH^•^, which directly quenches Fenton-generated hydroxyl radicals (17). The contribution of singlet oxygen and hydroxyl radicals to aBL killing of S. aureus, E. coli, and P. aeruginosa is displayed in Fig. 6. In the presence of NaN_3_, aBL killing of P. aeruginosa was significantly reduced. After 68.7 J/cm^2^ of aBL exposure, lower bacterial killing (around 3-fold) was observed in the presence of NaN_3_ (1.68 ± 0.29 log_10_) than in untreated control (5.38 ± 0.49 log_10_, *P* = 0.002). Although less pronounced, thiourea also promoted bacterial reduction (3.76 ± 0.50 log_10_) compared to control at 68.7 J/cm^2^ of aBL exposure.

For S. aureus, statistically significant differences in CFU reduction were only observed between NaN_3_ and untreated control groups at aBL exposure of 137.4 J/cm^2^ (2.46 ± 0.75 log_10_ X 4.54 ± 1.22 log_10_, *P* = 0.04). Thiourea also promoted a photoprotective effect against aBL exposure in S. aureus (1.88 ± 0.61 log_10_) even though no statistically significant differences were detected. Regarding E. coli, we observed photoprotective effects of NaN_3_ and thiourea (4.63 ± 0.57 log_10_ and 2.60 ± 1.21 log_10_, respectively) compared to the control (5.67 ± 0.57 log_10_ of killing) but with no statistically significant differences among groups.

### aBL induced DNA damages in S. aureus and E. coli, but not P. aeruginosa.

**FIG 7:**
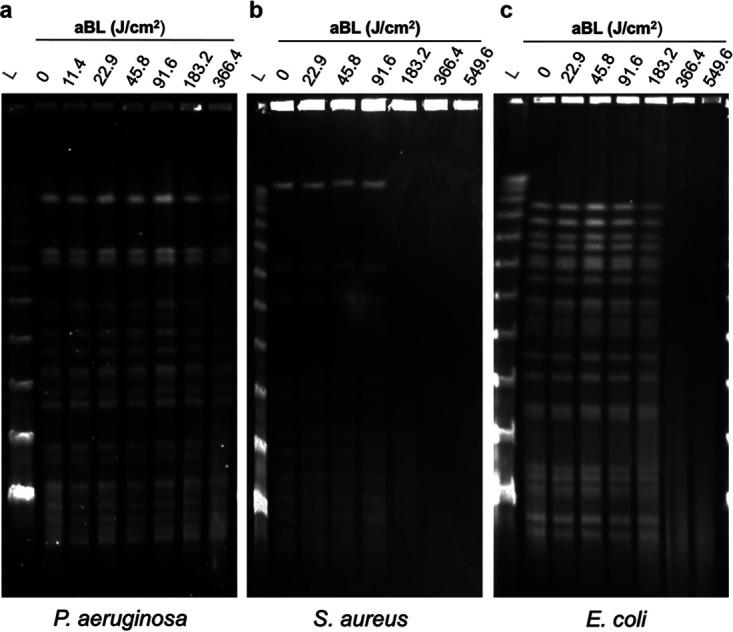
Analysis of DNA damage assayed by pulsed-field gel electrophoresis (PFGE) using the whole genome of P. aeruginosa (a), S. aureus (b), and E. coli (c). Bacterial samples were exposed to increasing radiant exposure of aBL (i.e., 0, 11.45, 22.9, 45.8, 91.6, 183.2, 366.4, and 549.6 J/cm^2^), followed by DNA extraction. For DNA cleavage, specific restriction endonucleases were used for each microorganism: *Xbal*, *BcuI*, and SmaI for E. coli, P. aeruginosa, and S. aureus, respectively. The reference molecular weight ladders for pulsed-field gel electrophoresis are identified by (L).

To test our hypothesis that aBL exposure induces DNA damage in bacteria, we assessed the degradation of DNA following different radiant exposures of aBL (Fig. 7). First, we observed no DNA degradation for P. aeruginosa, even for the highest aBL radiant exposure (366.4 J/cm^2^), able to kill 100% of bacteria (LD_100_ = 117.2 J/cm^2^). Second, S. aureus seems to be more susceptible than E. coli to DNA damage. Indeed, aBL exposures ≥183.2 J/cm^2^, which is sufficient to kill over 3 log_10_ CFU, (LD_99.9_ = 158.99 J/cm^2^), promoted significant DNA degradation in S. aureus whereas E. coli needed to be exposed to radiant exposures ≥366 J/cm^2^ (higher than LD_99.9999_ = 343.3 J/cm^2^).

### Bacterial proteins are susceptible to degradation by aBL at high radiant exposures.

**FIG 8:**
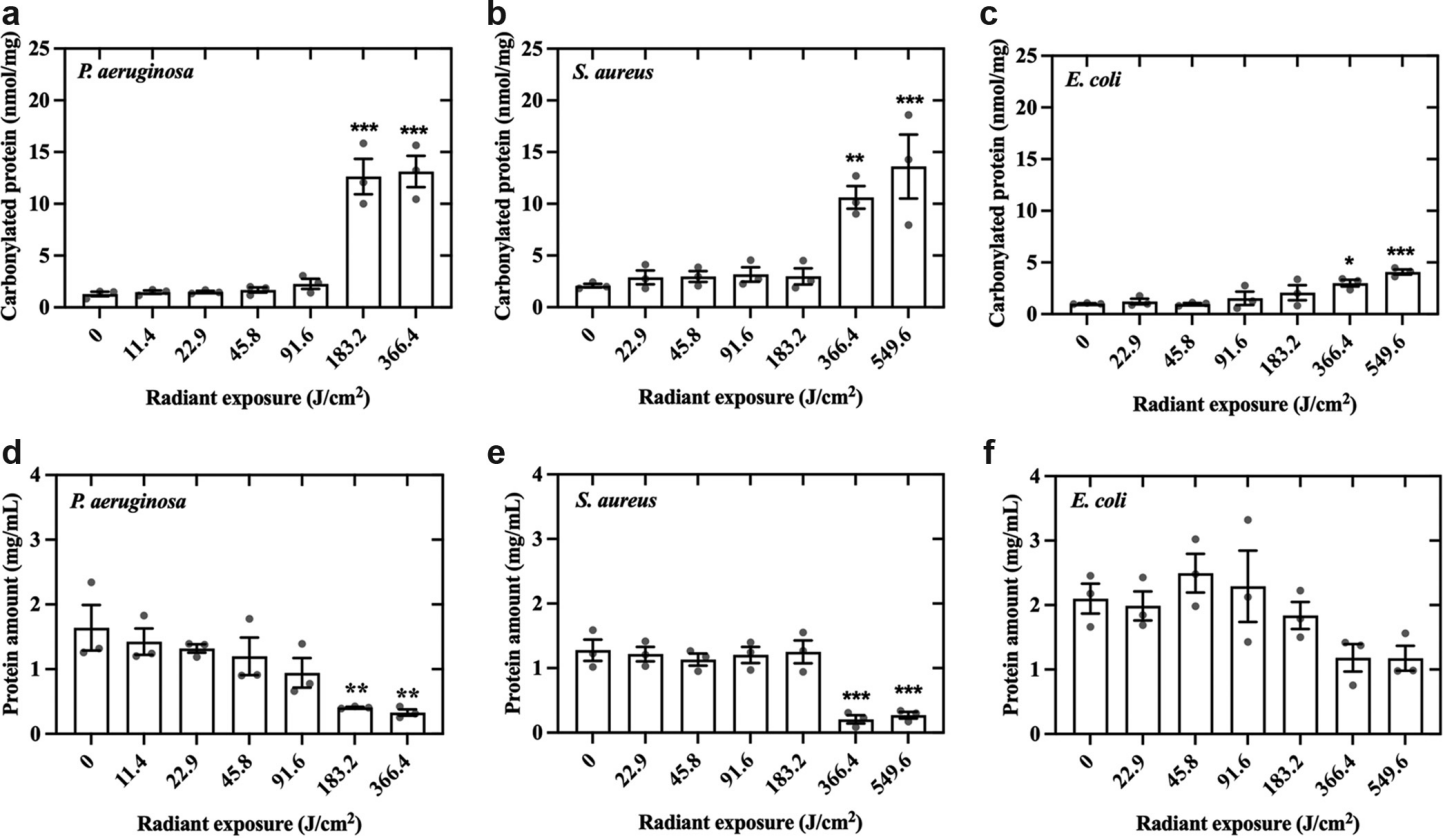
Effects of aBL on proteins in P. aeruginosa, S. aureus, and E. coli assessed by carbonyl protein quantification (a, b, and c) and total protein amount (d, e, and f). Symbols (•) represent the mean of three technical replicates. Data are presented as scatterplots with bar ± SEM. *, *P* < 0.05; **, *P* < 0.01; ***, *P* < 0.001 compared to unirradiated control.

The degradation of bacterial proteins is exhibited in Fig. 8. Protein carbonylation refers to the oxidation of protein side chains to form reactive ketones or aldehydes, and its quantification allows the evaluation of the extent of oxidative stress in the context of cellular damage (18). Regardless of the species, the reduction in total protein amount or the increase in carbonylated proteins depends on the aBL radiant exposures. For P. aeruginosa, a significant increase in the concentration of carbonylated proteins was observed after 183.2 J/cm^2^ of aBL (*P* < 0.0001). S. aureus and E. coli demonstrated reduced protein oxidation, compared to P. aeruginosa, with a significant increase in carbonylated protein concentrations only after 366.4 J/cm^2^ aBL (*P* = 0.002 and *P* = 0.02, respectively).

### aBL induced lipid peroxidation in P. aeruginosa, S. aureus, and E. coli.

**FIG 9:**
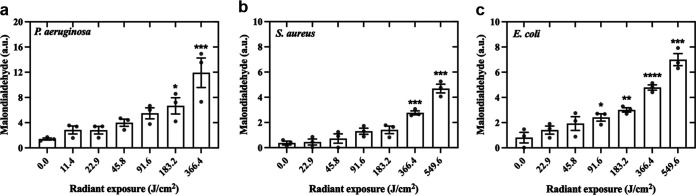
Effects of aBL on lipids in (a) P. aeruginosa, (b) S. aureus, and (c) E. coli assessed using malondialdehyde as a marker for lipid peroxidation. Symbols (•) represent the mean of three technical replicates. Data are presented as scatterplots with bar ± SEM. *, *P* < 0.05; **, *P* < 0.01; ***, *P* < 0.001 compared to the unirradiated control.

Regardless of the species, the higher the dose, the greater the lipid peroxidation, which is proportional to the number of malondialdehyde groups (Fig. 9). Statistically significant differences compared to control were observed for higher aBL exposures (183.2 and 366.4 J/cm^2^ for P. aeruginosa, and S. aureus, respectively). In contrast, lower doses of aBL (91.6 J/cm^2^) trigger significant lipid peroxidation in E. coli. Interestingly, levels of malondialdehyde (MDA) for P. aeruginosa are approximately 2-fold higher than for E. coli, regardless of the light dose.

### aBL resulted in cell membrane damage in P. aeruginosa and E. coli, but not S. aureus.

**FIG 10:**
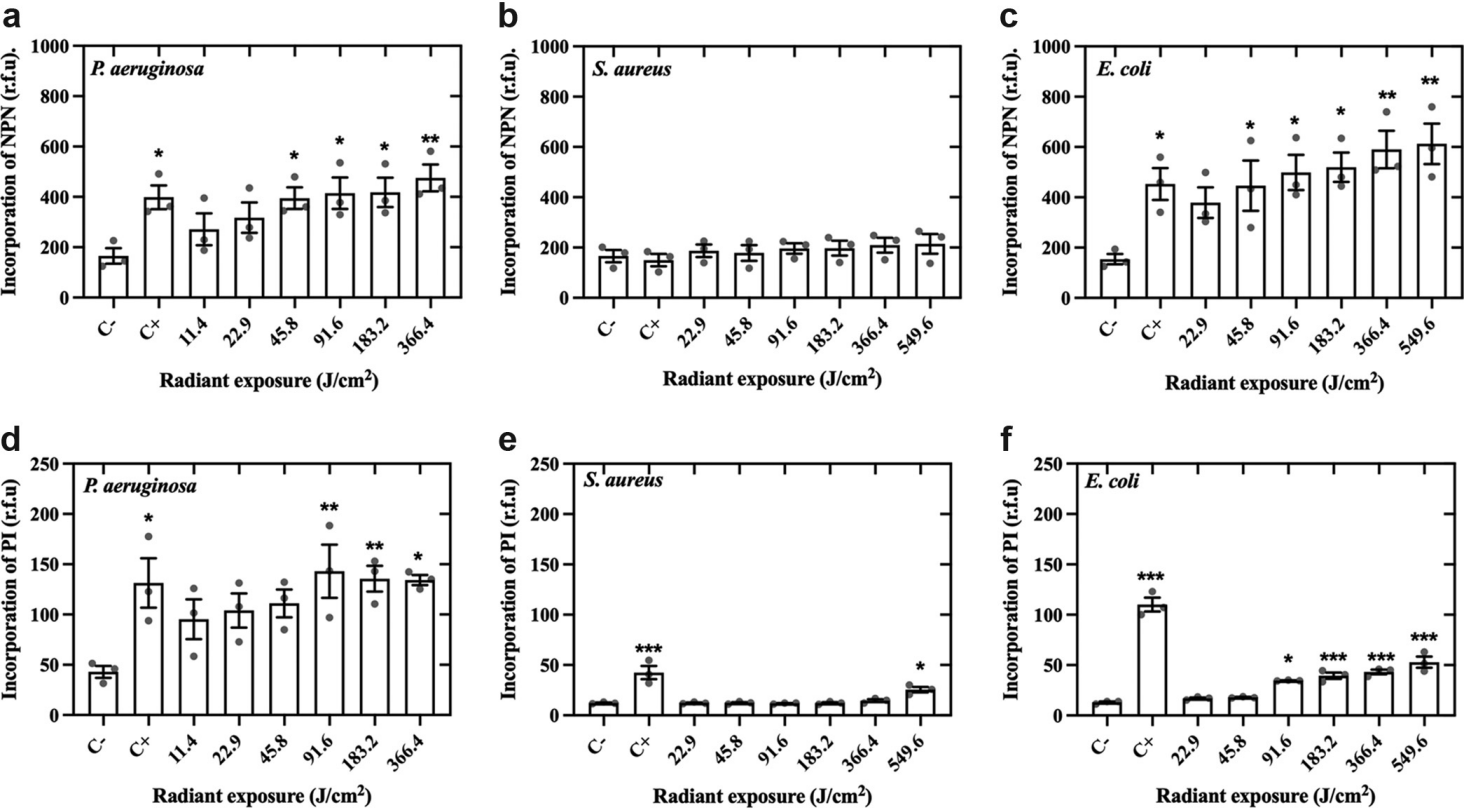
Effects of aBL on membrane permeabilization in P. aeruginosa, S. aureus, and E. coli assessed by N-phenyl-1-naphthylamine (NPN, a, b, and c) and propidium iodide (PI, d, e, and f). Symbols (•) represent the mean of three technical replicates. Data are presented as scatterplots with bar ± SEM. *, *P* < 0.05; **, *P* < 0.01; ***, *P* < 0.001 compared to the negative control (C−). Positive control (C+) for membrane damage samples were treated with ethanol at 70% for 15 min.

We evaluated changes to the integrity of the bacterial cell membrane using propidium iodide (PI) and N-phenyl-1-naphthylamine (NPN) (Fig. 10). For P. aeruginosa and E. coli, after 45.8 J/cm^2^ of aBL, we observed a significant increase in NPN incorporation, indicative of membrane damage (*P* < 0.01). Similarly, a significant increase in PI incorporation was observed after the aBL exposure of 91.6 J/cm^2^ (*P* < 0.01). In contrast, for S. aureus, the PI incorporation was only detected after aBL radiant exposure of 549.6 J/cm^2^ (*P* < 0.05), which was higher than LD_100_ = 397.7 J/cm^2^. On the other hand, NPN incorporation was unaltered, likely due to the structural differences in Gram-positive membranes.

## DISCUSSION

Herein, we investigated the targets involved in the aBL-mediated killing of bacteria for three clinically important bacterial species: P. aeruginosa, S. aureus, and E. coli. Previous studies have suggested that ROS generation by aBL exposure could lead to the damage of multiple nonspecific targets, including DNA, proteins, lipids, and the cell wall/membrane (11–13).

We found differences in the efficacy of aBL among species, especially when comparing P. aeruginosa to S. aureus or E. coli, which are noticeably more tolerant to aBL. The literature has pointed to porphyrins as essential intracellular chromophores that are excitable by aBL and produce ROS as a result (19–21). Therefore, a potential explanation for the different dose-response curves relies on the endogenous porphyrin concentration in each bacterial species. Thus, we quantified the endogenous porphyrins of these three species and observed that P. aeruginosa presents the highest concentration of porphyrins, which might explain its higher sensitivity to aBL. Interestingly, the porphyrin concentration in S. aureus was only 34% lower than that of P. aeruginosa, whereas the concentration in E. coli was 95% lower for P. aeruginosa. However, the killing observed between S. aureus and E. coli by aBL was rather similar, even though porphyrin concentration differed by 6.7-fold. This finding suggests that tolerance to aBL could not be a result of porphyrin concentration only. Indeed, Leanse et al. have reported that the presence of the powerful antioxidant staphyloxanthin, which is a carotenoid pigment in the S. aureus membrane, is responsible for aBL tolerance (22). Therefore, it is likely that, while porphyrin concentration is an important factor that drives aBL efficacy, other factors such as the presence or the synthesis of antioxidant molecules may be equally essential.

We then investigated the production of ROS following increasing aBL radiant exposures for each species. For P. aeruginosa and E. coli, we observed a direct dose-response on ROS production, even though the detected ROS was remarkably lower for E. coli. We assume that the lower amount of endogenous porphyrins in E. coli is responsible for this reduced ROS production. In contrast, ROS formation by S. aureus reaches a peak when exposed to 4 J/cm^2^. For higher radiant exposures, changes in ROS production were less pronounced. This could be explained by the antioxidant defenses of S. aureus, which seem to be faster triggered or more abundant than those of P. aeruginosa and E. coli. Indeed, NaN_3_ and thiourea equally photoprotected S. aureus from singlet oxygen and hydroxyl radical attack. In contrast, singlet oxygen and hydroxyl radical play an important role in the killing of P. aeruginosa and E. coli, respectively.

Following exposure of bacteria to LD_90_ and LD_99.9_ of aBL, we observed pronounced ultrastructural damages, which were expected due to ROS production (8, 23). Cell/wall membrane showed loss of integrity in the form of rupture (P. aeruginosa) and conformational changes (S. aureus and E. coli). These findings are corroborated by PI staining and NPN assays. For P. aeruginosa and E. coli, sublethal doses (<LD_90_) of aBL elicited significant PI and NPN incorporation, which suggests membrane permeabilization, as reported by other authors (11, 24). However, S. aureus showed more resistance to cell wall/membrane damage by aBL. In this case, we hypothesize that the LD_99.9_ induced a conformational change in the cell wall/membrane of S. aureus but with no impact on the membrane permeability.

Another interesting finding is related to the quantification of lipid peroxidation by measuring MDA. Although changes in peroxidation levels have been observed, high radiant exposures were necessary to induce lipid peroxidation, which matches the results reported by Wu et al. (13). We also observed that levels of lipid peroxidation for P. aeruginosa are two times higher than for E. coli (see Fig. 9). These data could explain the membrane rupture of P. aeruginosa observed by TEM.

According to previous studies (25, 26), ROS production is correlated to the presence and excitation of endogenous porphyrins. Our FLIM data might therefore be associated with the localization of endogenous porphyrins. To corroborate our hypothesis, we imaged fluorescence distribution inside bacterial cells by confocal FLIM using excitation at 405 nm and emission at wavelengths longer than 590 nm. This configuration basically selects porphyrin emission because, although flavins absorb around 405 nm, their emission does not go beyond 570 nm (27). We noticed that porphyrins are uniformly distributed throughout the bacterial cell. In addition, each bacterial species presented different fluorescence lifetimes (see Fig. 4). Although these microorganisms are known to have different concentrations of intracellular porphyrins (see Fig. 3), the concentration difference cannot explain the differences in the lifetime because the fluorescence decay does not depend on the fluorophore concentration (28). As the fluorescence lifetime of intracellular porphyrins has not been fully characterized yet, the different lifetime decays observed here should indicate either the presence of different porphyrins, or different supramolecular interactions with other molecules, such as lipids, proteins and nucleic acids, suggesting different targets for aBL.

Regarding DNA, we verified that the highest radiant exposures of aBL were able to induce DNA damage in S. aureus and E. coli. Indeed, E. coli required about 2-fold the dose necessary to completely degrade DNA compared to S. aureus. To our surprise, however, there was no evidence of DNA degradation in P. aeruginosa, even at radiant exposures necessary to kill 100% of bacteria (around 120 J/cm^2^). We suppose that the severe damage promoted by LD_99.9_ in the cell membrane of P. aeruginosa mitigated additional damage to the DNA because ROS lifetime is quite short. DNA is an extremely hardy molecule and sustained ROS production is likely required to elicit significant DNA degradation. On the other hand, aBL killing of S. aureus and E. coli did not result in membrane rupture, suggesting that ROS formation was sustained and contained inside the cell allowing DNA degradation.

Protein damage has also been suggested to occur as a result of aBL exposure (12). Therefore, we evaluated whether aBL could promote protein degradation. Our data showed that the total protein content of bacteria was reduced, which simultaneously increased protein carbonylation following high aBL exposures, regardless of the bacterial species. Walker et al. have suggested that protein damage through oxidation and carbonylation would be the first photodynamic effect induced by 405 nm in C. jejuni, given the high reaction constant of singlet oxygen with proteins (12). Other approaches to kill bacteria involving oxidative stress such as methylene-blue mediated photodynamic therapy, UV light, and ionizing radiation, can also trigger cell death by protein damage (29, 30).

It is well known that some ROS, such as singlet oxygen and hydroxyl radicals, are capable of oxidizing nearly all biological targets. However, the reaction triggers have minimal diffusion from their production site. Conversely, radicals less reactive diffuse over greater distances in the bacterial cell, being limited by physical barriers and charge interactions (31). Thus, damage in bacterial targets is based upon several factors, including the rate constant for the oxidant's reaction with the target, the location of the target relative to that of the oxidant, the occurrence of secondary damaging events, such as chain reactions or the occurrence of intra- and intermolecular reactions (29, 31). Repair mechanisms and the presence of antioxidants in the bacterial cell also influence the bacterial response (32).

It is important to highlight that, although we have compared species using ATCC strains as a standard of the field, sensitivity to aBL can also be strain-specific. Indeed, different strains of the same bacterial species show variable susceptibility to aBL depending on the strain's characteristics, such as phenotypic profile and endogenous pigments (9, 33). Thus, additional studies carried out on clinical isolates, as well as on polymicrobial communities, are welcome to improve the understanding of aBL targets.

In conclusion, our results clearly show that aBL induces significant antimicrobial effects on different bacterial species. However, we observed variable efficacy of aBL, which appears to be species-dependent and related to porphyrin concentration, antioxidant defenses, and ROS production. Our findings also suggest that the cell membrane is the preferential target of aBL for P. aeruginosa and E. coli because low radiant exposures are necessary to promote membrane damage. Additionally, although degradation of biomolecules has been detected for S. aureus following high doses, membrane permeabilization was only detected at doses above LD_100_. Taken together, these findings indicate that the primary targets of aBL strongly depend on the bacterial species.

## MATERIALS AND METHODS

### Bacterial strains and culture conditions.

We used three bacterial species acquired from the American-type culture collection (ATCC; Manassas, VA): S. aureus (Gram+, 25923), E. coli (Gram-, 25922), and P. aeruginosa (Gram-, 27853). These were cultured on brain heart infusion (BHI) agar or BHI broth overnight (18 h) and incubated at 37°C in a stationary or shaking incubator. Growth conditions were the same for the three bacterial species.

### Light source.

Bacteria were illuminated using a blue light emitting diode (LED) array (λ = 410 ± 10 nm, LEDbox, BioLambda, Brazil) to allow homogenous (>90%) exposure of microtiter plates. The irradiance was adjusted to 38.2 mW/cm^2^. The spectral emission was confirmed by using a UV-VIS spectrophotometer (Flame, Ocean Optics, USA).

### aBL killing of bacteria.

Stationary-phase bacteria were cultured in 10 mL of BHI and incubated overnight (18 h) with shaking (100 rpm). After incubation, bacteria were centrifuged at 7,378 × *g* for 10 min, washed twice, and suspended in phosphate-buffered saline (PBS; dibasic phosphate = 2.65 g/L, monobasic phosphate = 0.358 g/L, NaCl = 8.183 g/L, and milli-Q water; pH 7.4). The inoculum was standardized to approximately 1 to 2 × 10^9^ CFU (CFU)/mL by turbidity of the suspension with a spectrophotometer (λ = 625 nm; optical path = 1 cm; optical density = 0.667). Subsequently, 1 mL of the bacterial suspension was transferred to 12-well microtiter plates. All strains were subjected to the following radiant exposures: 22.9, 45.8, 68.7, 91.6, 114.5, 137.5, 160.4, 183.3, 206.2, and 229.1 J/cm^2^. The negative-control group (nonirradiated) remained in the dark during the total exposure time. Following each light dose of aBL, bacterial viability was determined by serial dilution, plating, and CFU/mL counting as described by Jett et al. (34). Survival fraction values were determined as CFU/mL averages, normalized with each respective nonirradiated group (N_0_/N; i.e., N_0_= initial microbial burden; N= final microbial burden) and converted into log_10_. The lethal dose (LD) for a 99.9 killing rate (i.e., 3 log_10_) was calculated according to the method described by Sabino et al. (35).

### Transmission electron microscopy to determine ultrastructural changes in bacteria following aBL exposure.

TEM was employed to visualize the ultrastructural changes that result from aBL exposure. Radiant exposures found to kill 90 or 99.9% (LD_90_ and LD_99.9_, respectively) of each bacterial species (E. coli, S. aureus, and P. aeruginosa) were selected for imaging using TEM. Additionally, untreated (negative) control samples ran in parallel. After aBL exposures, bacterial cells were harvested by centrifugation, fixed in 2% glutaraldehyde, and kept overnight at 4°C. The samples were then centrifuged at 10,000 g for 10 min and washed in 0.1 M sodium cacodylate buffer (pH 7.2) twice. Pellets were postfixed in 2% osmium tetroxide in sodium cacodylate buffer for 8 h. Samples were then serially dehydrated in ethanol, and embedded in Spurr resin (Tousimis, USA). Ultrathin slices (<100 nm) were cut by diamond blades of an ultramicrotome (Leica, Germany). Samples were placed on a 200-mesh copper grid, contrasted with 4% uranyl acetate for 5 min, and 0.3% lead citrate for 15 min. The observation and capture of images were performed in a transmission electron microscope (JEOL, model 1010, Tokyo, Japan), in increments of up to 100,000×, operating at 80 kV. Images obtained by TEM were selected based on the most common cell damage observed.

### Porphyrin detection and quantification by ultraperformance liquid chromatography in the absence of aBL.

Overnight cultures of bacteria grown in 60 mL of BHI broth (20 mL cultures × 3 in 50 mL tubes) were pelleted by centrifugation in 7,378 × *g* for 10 min, resuspended into 1-mL extraction solution (ethanol, dimethyl sulfoxide, acetic acid, 80:20:1; vol/vol/vol) and stored at −80°C. After 24 h of incubation, cells were disrupted by an ultrasonic bath at 40 kHz (Bransonic 2510R-MT, Danbury, CT) and the porphyrin-containing supernatant was recovered for quantification by ultraperformance liquid chromatography (UPLC, Waters Acquity UPLC system) analyses as performed previously (8). In brief, chromatographic marker kits that enable the detection of uroporphyrin, heptaporphyrin, hexaporphyrin, pentaporphyrin, coproporphyrin, and protoporphyrin IX were used, and the results were compared with chromatographs produced by standard porphyrins.

### FLIM to identify fluorophore distribution inside the bacterial cell in the absence of aBL.

Spatial localization and fluorescence lifetime of endogenous fluorophores were observed through fluorescence-lifetime imaging confocal microscopy. Overnight cultures of bacteria were harvested by centrifugation, washed twice with sterile PBS, and adjusted in PBS to OD_625_ = 0.667 (approximately 1 to 2 × 10^9^ CFU/mL). The time-resolved fluorescence detection was carried out by a confocal laser scanning microscope (MicroTime 200, PicoQuant), equipped with laser excitation at 405 nm, photomultiplier, 590 (36) bandpass filter, short pulse (<1 ns) and ×60 magnification to analyze samples at a super-resolution level (i.e., below the light diffraction limit). The images and data were analyzed using SymPhoTime 64 software (PicoQuant, Germany).

### Detection of general reactive oxygen species during aBL exposure.

Intracellular ROS production was quantified with the use of the bacterial permeant probe 2′, 7′-dichlorofluorescein diacetate (DCFH-DA; D6883, Sigma-Aldrich) as described previously (13). Under normal conditions, DCFH-DA does not emit a fluorescent signal; however, once oxidized it becomes increasingly fluorescent. In this study, DCFH-DA at a final concentration of 20 μM was applied to bacteria (10^9^ CFU/mL), which were incubated at 25°C for 30 min to allow permeation of the probe. Samples were then exposed to aBL over increasing radiant exposures of 0, 0.5, 1, 2, 4, 8, 16, 32, and 64 J/cm^2^, and then immediately transferred to 96-well plates. Following each aliquot of aBL, fluorescence was measured every 5 min (λ_exc_ = 504 nm and λ_em_ 529 nm) on a microplate reader (Spectramax M4, Molecular Devices, Sunnyvale, CA) for a total of 1 h.

### Effects of ROS scavenging on aBL efficacy.

To explore the relative contributions of specific ROS on the efficacy of aBL, we exploited the use of specific ROS scavengers: sodium azide (NaN_3_; singlet oxygen scavenger) (37) or thiourea (hydroxyl radical scavenger) (16), which were previously shown to be produced by bacteria following aBL illumination (17). Initially, stationary-phase bacteria were resuspended in PBS at 1 to 2 × 10^9^ CFU/mL, and NaN_3_ or thiourea were added to a final concentration of 10 mM and 150 μM, respectively (16, 37). The bacterial suspension (1 mL) was subsequently transferred to a 12-well microtiter plate, and aBL radiant exposures were applied based on tolerance observed with each respective species. For S. aureus and E. coli, the following aBL exposures were applied: 0, 45.8, 91.6, and 137.4 J/cm^2^, and P. aeruginosa was exposed to 0, 22.9, 45.8, and 68.7 J/cm^2^ of aBL. For negative control, all inocula were maintained in PBS under equivalent conditions. The aBL exposure and CFU/mL quantification were performed according to the previously described.

### Elucidating DNA damage following aBL exposure.

Pulsed-field gel electrophoresis (PFGE) analysis was performed to assess potential DNA breakages induced by aBL. Bacterial suspensions of S. aureus, E. coli, and P. aeruginosa were prepared as described above and exposed to increasing aBL radiant exposures of 0, 11.45, 22.9, 45.8, 91.6, 183.2, 366.4, and 549.6 J/cm^2^. Following treatment, E. coli and P. aeruginosa suspensions were pelleted by centrifugation (7,378 × *g* for 10 min) and resuspended in 200 μL of TE I buffer (Tris HCl 10 mM, EDTA 1 mM, pH 7.5). For S. aureus, an extra step, including 4 μL lysostaphin (L-7386; Sigma) stock solution (1 mg/mL in sodium acetate 20 mM, pH 4.5) was added. Samples (8 μL) were added into 320 μL of pulsed-field agarose solution (1%; Bio-Rad, USA) at 55 to 60°C and casted into a plug mold (36). Following solidification, E. coli and P. aeruginosa sample plugs were removed and placed into a lysis buffer (EDTA 50 mM, Tris-HCl 50 mM, SDS 1%, Sarkosyl 1%, proteinase K 0.1 mg/mL, pH 7.5). S. aureus sample plugs were immersed into a modified lysis buffer (EDTA100 mM, Tris-HCl 6 mM, NaCl 1 M, Brij-58 0.5%, sodium deoxycholate 0.2%, N-lauroylsarcosine 0.5%, pH 7.5). All samples were incubated at 55°C for 2 h. After lysis, plugs were washed twice in milli-Q water and four times in TE I at 15-min intervals, at 55°C.

For DNA cleavage, specific restriction endonucleases were used for each microorganism: *Xbal* (ER0682, Thermo Fisher Scientific, USA), *BcuI* (ER1251, Thermo Fisher Scientific, USA), and SmaI (ER0662, Thermo Fisher Scientific, USA) for E. coli, P. aeruginosa and S. aureus, respectively. Plugs were transferred into tubes containing 10 U restriction enzyme in its proper buffer and incubated at 37°C for 18 h. Following the incubation, plugs and DNA ladder (Lambda PFG Ladder, New England Biolabs) were inserted into 1% agarose gel and immersed in TBE buffer (Tris-borate 45 mM, EDTA 1 mM, pH 8.0). Electrophoresis was performed using the Chef Mapper apparatus, Bio-Rad, and configured to 6 V/cm at 14°C for 22 h, with pulse intervals between 3.51 s and 30.82 s. DNA was stained with ethidium bromide (1 μg/mL) and visualized at UV light (Epi Chemi II Darkroom, UVP Bioimaging Systems).

### Assessing protein damage in bacteria by aBL through total protein and carbonyl quantification.

To determine whether bacterial proteins were oxidized following exposure to aBL, we quantified total protein content as well as total carbonylated protein content. Stationary-phase bacterial suspensions were prepared to 1 to 2 × 10^9^ CFU/mL and exposed (1 mL; 12-well plates) to increasing aBL exposures of 11.4, 22.9, 45.8, 91.6, 183.2, 366.4, and 549.6 J/cm^2^. For each bacterial species, a total of 12 technical replicates were performed and then pooled together, to collect enough bacteria for protein extraction. The bacterial pellets were then recovered by centrifugation and resuspended in a 60 μL sample diluent from the OxiSelect Protein Carbonylation Fluorimetric Assay (STA-307; Cell Biolabs Inc., USA). Cell disruption was achieved by using a probe sonic dismembrator (Model 100, Fisher Scientific, USA) for 5 s. Five cycles of pulsing-cooling were performed. The supernatants were recovered by centrifugation at 10,000 × *g* for 5 min at 4°C. A total of 50 μL were used as input and protein carbonylation analysis was performed according to the manufacturer’s instructions. In parallel, the total protein present in the samples was measured using a BCA Protein Assay (Thermo Fisher Scientific, USA) to normalize carbonyl content (nmol/mL) by protein amount (mg/mL).

### Lipid peroxidation following aBL exposure.

The measurement of the end products of lipid peroxidation, MDA, was performed to assess possible damages induced by aBL exposure. The stationary phase of bacterial suspensions was adjusted to a bacterial concentration of approximately 1 to 2 × 10^9^ CFU/mL and exposed to increasing exposures of aBL at 0, 11.4, 22.9, 45.8, 91.6, 183.2, 366.4, and 549.6 J/cm^2^. Following aBL exposure, the quantification of MDA was performed using an OxiSelect TBARS Assay (STA-330; Cell BioLabs Inc., USA), according to the manufacturer's instructions. The fluorescent reaction was measured at λ_exc_ = 540 nm and λ_em_ 590 nm.

### Membrane/cell wall damage following aBL exposure.

To determine if the bacterial membrane/cell wall was damaged as a result of aBL exposure, two methods were employed to quantify damage, including the use of PI and NPN (38, 39). PI is a fluorochrome that binds to DNA strands but cannot pass through a healthy cytoplasmic membrane. NPN is a nonpolar probe that fluoresces strongly in phospholipid environments like the internal face of the outer membrane (OM) of Gram-negative bacteria (39, 40). Bacterial samples (as described previously) were exposed to aBL at the exposures previously calculated to reach LD_50_, LD_90_, and LD_99.9_ killing rates. Two comparative controls were included for each species: positive membrane damage control (70% ethanol for 15 min, which is known to lyse bacterial cells [41], and negative control, untreated sample). For the PI uptake assay, samples were distributed into 96-well plates and incubated with PI (50 μg/mL; BD Pharmingen, USA) in dark for 15 min. The PI incorporation into the membrane was measured using a plate reader spectrophotometer (SpectraMax M4; Molecular Devices, USA) at λ_ex_ = 520 nm and λ_em_ 620 nm. Using a similar method, for outer membrane assay, samples were incubated with NPN 15 μM (Sigma-Aldrich, USA). The fluorescent NPN incorporation was measured at λ_ex_ = 350 nm and λ_em_ 415 nm.

### Statistical analyses.

All experiments with quantitative data were carried out in three replicates and performed on three separate days. Data are presented as means and standard error of the mean (SEM). Statistical analysis was performed by using one- or two-way analysis of variance (ANOVA) with Tukey as posttest comparisons. The analysis was performed by Prism 9.0 software (GraphPad, USA). Values of *P* < 0.05 were considered statistically significant.
