# Supplementary material for: New Insights into the Bacterial Targets of Antimicrobial Blue Light
Source: Microbiol Spectr. 2023 Feb 21;11(2):e02833-22. doi: 10.1128/spectrum.02833-22 (PMC10101057; doi:10.1128/spectrum.02833-22)

Supplementary material

Figure 1. Representative transmission electron micrographs illustrating aBL-induced ultrastructural damages in *P. aeruginosa.* Bars: 500 nm.

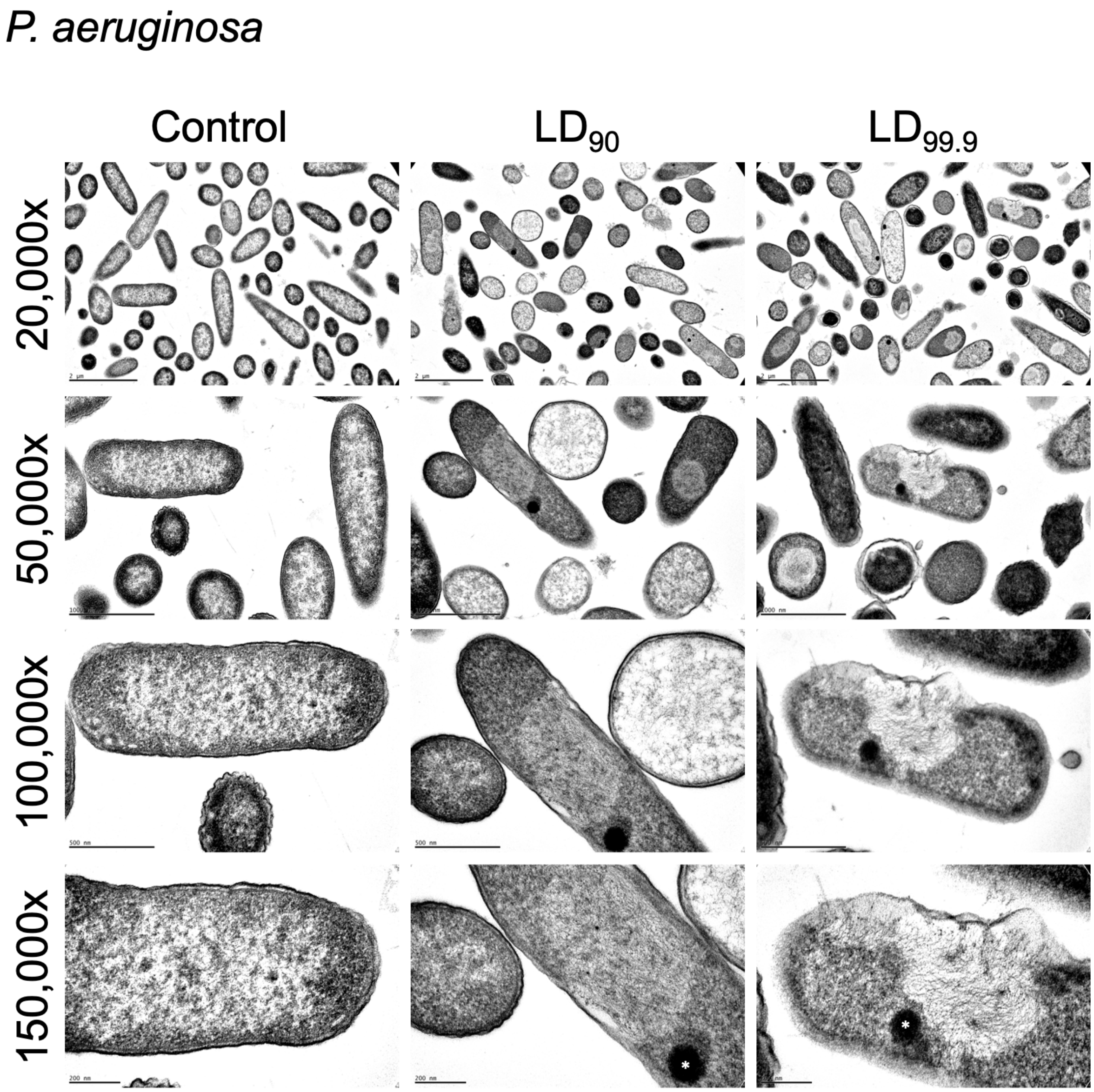

Figure 2. Representative transmission electron micrographs illustrating aBL-induced ultrastructural damages in *S. aureus*. Bars: 500 nm.

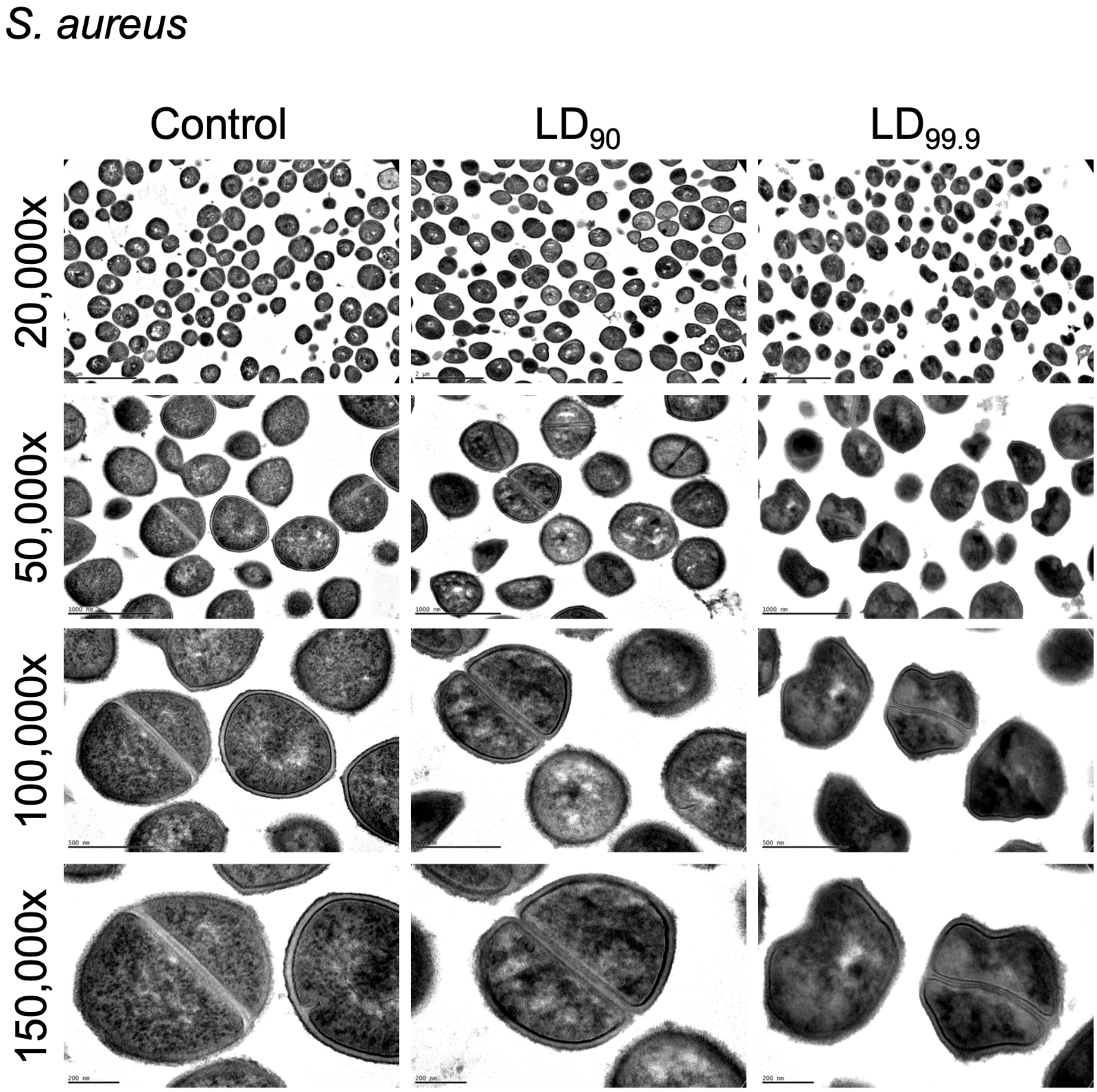

Figure 3. Representative transmission electron micrographs illustrating aBL-induced ultrastructural damages in *E. coli.* Bars: 500 nm.

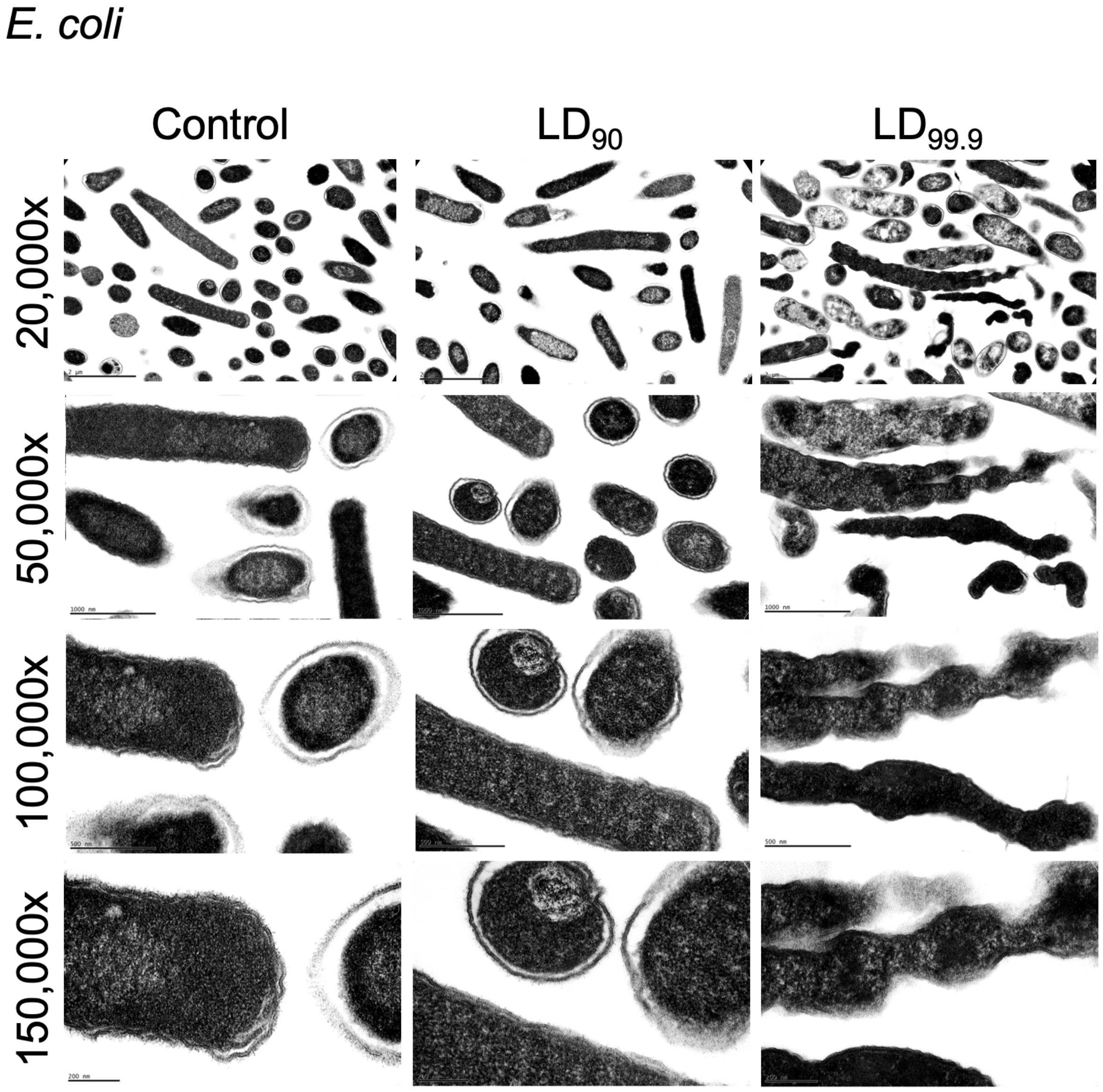

Supplement: Supplemental file 1 — Fig. S1 to S3. Download spectrum.02833-22-s0001.pdf, PDF file, 5.3 MB [file spectrum.02833-22-s0001.pdf]
